# Is State Recognition in Sight?

**Published:** 1916-02-26

**Authors:** 


### THE MATRONS' AND SISTERS' DEPARTMENT.

### IS STATE RECOGNITION IN SIGHT?

VOLUNTARY effort in the past has sometimes accomplished great triumphs, little, if anything, short of miracles. The promise of the moment, for which there is the soundest hope, is that it will in the near future prove strong enough to correct the evils that have developed in the nursing profession, by the united effort of its members through the College of Nursing, when State recognition will follow as a matter of course. We have strong reason for believing that such a unanimous effort will be secured by taking powers in the legal constitution incorporating the College to set up definite standards of education, examination, certification, and registration, with other important matters insisted upon, including the establishment of a register of all trained nurses throughout the United Kingdom who have obtained certificates of training and proficiency in nursing from a recognised nursing school. Such are some of the necessary preliminaries to secure by Act of Parliament an adequate recognition by the State. This being so, and we give our readers the assurance that we have satisfied ourselves that it is in fact the precise position at which affairs have arrived at the moment, we venture to invite all who have the best interests of British nursing and British nurses at heart, including the broader-minded registrationists, to send in their names to the Editor in support of such a scheme as the proper basis for the formation of the College of Nursing. Further, we have satisfied ourselves that there is every hope that the scheme of the College of Nursing, when it is legally authorised and finally settled, will be found to cover the whole field of nursing by the provision of a definite standard of education, protected by legal authority. It should enable every working nurse who is really efficient to prove her fitness, and so obtain status and protection for herself in the discharge of her important duties on behalf of the sick.

